# High resolution depth distribution of *Bacteria*, *Archaea*, methanotrophs, and methanogens in the bulk and rhizosphere soils of a flooded rice paddy

**DOI:** 10.3389/fmicb.2015.00639

**Published:** 2015-06-25

**Authors:** Hyo Jung Lee, Sang Eun Jeong, Pil Joo Kim, Eugene L. Madsen, Che Ok Jeon

**Affiliations:** ^1^Department of Life Science, Chung-Ang UniversitySeoul, South Korea; ^2^Division of Applied Life Science, Gyeongsang National UniversityJinju, South Korea; ^3^Department of Microbiology, Cornell UniversityIthaca, NY, USA

**Keywords:** methanogen, methanotroph, flooded rice paddy, depth distribution, methane, bulk and rhizosphere soil

## Abstract

The communities and abundances of methanotrophs and methanogens, along with the oxygen, methane, and total organic carbon (TOC) concentrations, were investigated along a depth gradient in a flooded rice paddy. Broad patterns in vertical profiles of oxygen, methane, TOC, and microbial abundances were similar in the bulk and rhizosphere soils, though methane and TOC concentrations and 16S rRNA gene copies were clearly higher in the rhizosphere soil than in the bulk soil. Oxygen concentrations decreased sharply to below detection limits at 8 mm depth. Pyrosequencing of 16S rRNA genes showed that bacterial and archaeal communities varied according to the oxic, oxic-anoxic, and anoxic zones, indicating that oxygen is a determining factor for the distribution of bacterial and archaeal communities. Aerobic methanotrophs were maximally observed near the oxic-anoxic interface, while methane, TOC, and methanogens were highest in the rhizosphere soil at 30–200 mm depth, suggesting that methane is produced mainly from organic carbon derived from rice plants and is metabolized aerobically. The relative abundances of type I methanotrophs such as *Methylococcus*, *Methylomonas*, and *Methylocaldum* decreased more drastically than those of type II methanotrophs (such as *Methylocystis* and *Methylosinus*) with increasing depth. *Methanosaeta* and *Methanoregula* were predominant methanogens at all depths, and the relative abundances of *Methanosaeta*, *Methanoregula*, and *Methanosphaerula*, and GOM_Arc_I increased with increasing depth. Based on contrasts between absolute abundances of methanogens and methanotrophs at depths sampled across rhizosphere and bulk soils (especially millimeter-scale slices at the surface), we have identified populations of methanogens (*Methanosaeta*, *Methanoregula*, *Methanocella*, *Methanobacterium*, and *Methanosphaerula*), and methanotrophs (*Methylosarcina, Methylococcus, Methylosinus*, and unclassified *Methylocystaceae*) that are likely physiologically active *in situ*.

## Introduction

Methane (CH_4_) is the second most important greenhouse gas in the atmosphere, after carbon dioxide. It is produced by methanogens in the biosphere, but a large part of the released methane is metabolized by methanotrophs before it reaches the atmosphere (Bridgham et al., 2013). Therefore, methane emission from the biosphere into the atmosphere is mainly determined by methanogenic and methanotrophic activities, and numerous prior studies have focused on methanogens and methanotrophs to gain a better understanding of methane metabolism in the biospheric carbon cycles (Semrau et al., 2010; Bridgham et al., 2013; Costa and Leigh, 2014). Among methanogenic groups, members of the orders *Methanomicrobiales*, *Methanobacteriales*, *Methanococcales*, *Methanosarcinales*, and *Methanocellales* have been identified from rice paddies (Sakai et al., 2009; Ma et al., 2012; Lee et al., 2014; Liu et al., 2015). Although anaerobic methanotrophs such as the anaerobic methanotrophic archaea (ANME), “*Candidatus* Methylomirabilis oxyfera,” and “*Candidatus* Methanoperedens nitroreducens” have recently been reported as putatively important players in methane oxidation (Ettwig et al., 2010; Biddle et al., 2012; Haroon et al., 2013; Shen et al., 2014), it has been suggested that methane is metabolized mainly by aerobic methanotrophs in rice paddies (Groot et al., 2003; Ma et al., 2010, 2013; Lee et al., 2014). Based on their physiological characteristics and phylogeny, the aerobic methanotrophs are generally divided into two groups, types I and II, corresponding to the family *Methylococcaceae* (*Gammaproteobacteria*) and the families *Methylocystaceae* and *Beijerinckiaceae* (*Alphaproteobacteria*), respectively (Semrau et al., 2010; Lüke and Frenzel, 2011). In addition, atypical methanotrophs belonging to the family *Methylacidiphilaceae* of the phylum *Verrucomicrobia* were reported to be aerobic (Op den Camp et al., 2009).

Rice, one of the most important crop plants worldwide with a total cultivation area of 155 million hectares, is traditionally grown under flooded or wet conditions during most of the cultivation period (Ma et al., 2010). Because the waterlogged soil of rice paddies provides ideal conditions for methanogenesis, rice field is considered an important anthropogenic methane source accounting for 5–19% of the global methane emission to the atmosphere (IPCC, 2007; Ma et al., 2010). Methane emission from rice paddies is the result of complicated processes, including hydrolysis and fermentation of organic matter, methanogenesis, and methane transport and oxidation, which involve complex consortia of hydrolytic, fermenting, methanogenic, and methanotrophic microorganisms (Liesack et al., 2000; Conrad, 2002, 2007). Previous studies have shown that different methanotrophic and methanogenic taxa display varying sensitivities and responses to oxygen concentrations (Gilbert and Frenzel, 1998; Yuan et al., 2009, 2011; Krause et al., 2010; Ma et al., 2012; Reim et al., 2012). Indeed, the abundance and distribution of methanogenic and methanotrophic communities both respond to and create the physical and chemical gradients that occur with depth in flooded rice paddy soils (Lüdemann et al., 2000; Noll et al., 2005; Krause et al., 2010; Reim et al., 2012; Ma et al., 2013).

Because it is well known that organic matter derived from detritus and root exudates of rice plants is an important driver of methane production and that oxygen influx occurs through the aerenchyma of rice roots, the abundance and structure of the methanogenic and methanotrophic communities along a depth gradient are expected to differ between the bulk and rhizosphere soils of planted and unplanted rice paddies. However, previous studies of microbial communities in rice paddies have largely relied upon terminal-restriction fragment length polymorphism (T-RFLP) analysis of 16S rRNA genes (Lüdemann et al., 2000; Noll et al., 2005; Reim et al., 2012). Although T-RFLP has been successfully used by some research groups to analyze methanogenic and methanotrophic communities (Ma and Lu, 2011; Ma et al., 2013), T-RPLP can deliver only limited insights into complex communities. Pyrosequencing is a more powerful technique to explore the composition of microbial communities in natural habitats (Roesch et al., 2007), and recently pyrosequencing has been used to analyze methanogen and methanotroph communities in rice paddies (Lee et al., 2014; Lüke et al., 2014; Breidenbach and Conrad, 2015). However, it has not yet been applied to samples gathered at millimeter resolution.

In this study, we combined the millimeter-scale sampling with pyrosequencing-based community characterization of communities, especially methanotrophs and methanogens, in bulk and rhizosphere soils of a planted rice paddy. Furthermore, we determined the oxygen, methane, and total organic carbon (TOC) concentrations in the corresponding depths. Thus, the findings reported here expand the current knowledge and understanding of methane production, oxidation, and transport in rice paddies.

## Materials and methods

### Rice paddy description and soil sampling along a depth gradient

Soil cores were samples from a rice paddy located in Sacheon, South Korea (35°10′90″N, 128°11′84″E). The rice paddy has a silt loam soil texture (20% clay, 55% silt, 25% sand) and has been tilled once a year for the last several decades. All farming practices, including tillage, transplanting of the Korean rice cultivar Dongjinbyeo (*Oryza sativa*, Japonica type), water irrigation, and chemical fertilization, were performed according to the same procedures described previously (Lee et al., 2014). Soil core sampling in the flooded rice paddy was performed on September 3, 2013, approximately 90 days after rice-transplanting (flowering and heading stages of rice growth), as methane emission reaches a maximal level during this phase of the rice cultivation period (Lee et al., 2014). Prior to the soil core sampling, oxygen concentrations along a depth gradient in the bulk and rhizosphere soils of the rice paddy were measured *in situ* using the Fibox 3 LCD trace system with the Pst3 sensor (PreSens GmbH, Rogensburg, Germany). Soil cores (3-cm diameter and 45-cm length) were collected from the bulk soil (at the mid-diagonal point separating individual plants placed at 30 × 15 cm spacings) and from the rhizosphere soil (~3 cm from the base of rice plants) within the flooded rice paddy (Supplementary Figure S1), using polyvinyl chloride soil core samplers sealed with rubber stoppers.

The sampled tubes were immediately frozen in a dry ice/ethanol bath, and then stored at −80°C until further analysis.

For the analysis of the microbial communities and methane concentrations along a depth gradient, 1.0-mm-thick sections were cut from the surface of the frozen soil cores to a 10-mm depth using a microtome cryostat (HM 505E, Microm, Germany) as described previously (Reim et al., 2012). In addition, soil samples corresponding to 30, 60, 100, 200, 300, and 400 mm depth were cut from the frozen soil cores using the microtome cryostat. Half of each frozen soil subsample was transferred into a 10-ml serum bottle, which was immediately sealed with rubber stoppers for the analysis of the methane concentration. The remaining half of soil subsamples were stored at −80°C until the analysis of the microbial community structure and abundance.

### Analysis of methane and TOC concentrations

To measure methane concentrations along a depth gradient in the rice paddy, the 10-ml serum bottles containing the soil samples were warmed to 25°C and shaken for 1 min after injection of 1 ml of distilled water (DW). Headspace gas samples (0.5 ml) were taken from the serum bottles using a gas-tight syringe (Hamilton, USA) and methane concentrations were measured with a 6890N gas chromatograph (GC, Agilent Technologies, USA) equipped with a flame-ionization detector and an HP-5 capillary column (0.32 mm × 30 m, 0.25 μm film thickness, J & W Scientific, USA) as described previously (Herman and Roberts, 2006). The methane concentrations in the rice paddy soil were calculated on a fresh weight basis by measuring the weights of the soil subsamples used for methane analysis. The measurements were performed in triplicate.

To measure TOC concentrations along a depth gradient, seven soil samples corresponding to 0–10, 25–35, 55–65, 95–105, 195–205, 295–305, and 395–405 mm depths in the bulk and rhizosphere soils of the rice paddy were collected from the frozen soil cores using a small handsaw. Twenty-five milliliters of DW were added to 5 g of the moist soil samples and the mixtures were agitated for 60 min using a shaker at 250 rpm. The mixtures were centrifuged at 8000 rpm (~7200 g) for 10 min and the supernatants were filtered using 0.45-μm pore-size filters. The TOC concentrations of the filtrates were measured using a TOC analyzer (TOC-VCPH, Shimadzu, Japan). The TOC concentrations in the rice paddy were calculated on a dry weight basis by measuring the dry weight of the precipitates after centrifugation. The measurements were performed in triplicate.

### Quantitative real-time polymerase chain reaction (qPCR)

To estimate the bacterial and archaeal abundances along a depth gradient in the rice paddy, qPCR was performed as described previously with some modifications (Lee et al., 2012, 2014). Briefly, genomic DNA was extracted from the remaining half of frozen soil subsamples using a Fast DNA spin kit (MP Biomedicals, Solon, OH) according to the manufacturer's instructions. Two qPCR primer sets, bac1114F (5′-CGG CAA CGA GCG CAA CCC-3′)/bac1275R (5′-CCA TTG TAG CAC GTG TGT AGC C-3′) and arch349F (5′-GYG CAS CAG KCG MGA AW-3′)/arch806R (5′-GGA CTA CVS GGG TATC TAA T-3′), were used to amplify the 16S rRNA genes of *Bacteria* and *Archaea*, respectively (Takai and Horikoshi, 2000; Denman and McSweeney, 2006). The qPCR amplifications were performed in triplicate and gene copy numbers were estimated by using two standard curves generated from pCR2.1 vectors (Invitrogen, USA) carrying bacterial (*Chloroflexi*) and archaeal (a methanogenic archaeon) 16S rRNA genes as described previously (Jung et al., 2011; Lee et al., 2012). Bacterial and archaeal 16S rRNA gene copy numbers in the rice paddy were calculated on a fresh weight basis by measuring the weight of the soil samples used for DNA extraction.

### Analysis of the bacterial and archaeal community structure using pyrosequencing

For the analysis of bacterial and archaeal community structures along a depth gradient in the rice paddy, composite genomic DNA samples were prepared by mixing equal amounts of the genomic DNA extracted from the same depths of three different cores. The hypervariable regions of bacterial and archaeal 16S rRNA genes were amplified using primer sets Bac9F/Bac541R and Arc344F/Arc927R with unique 7–11 mer barcode sequences, respectively (Supplementary Table S1). The PCR products were pooled for pyrosequencing using a 454 GS-FLX Titanium system (Roche, Mannheim, Germany) at Macrogen (Seoul, Korea) as described previously (Lee et al., 2014).

The pyrosequencing data were processed using RDPipeline (http://pyro.cme.msu.edu; Cole et al., 2009). The pyrosequencing reads were sorted to the specific samples based on their unique barcodes, after which the barcode and primer sequences were trimmed. Reads with more than two undetermined nucleotides and/or read lengths shorter than 300 bp were excluded from subsequent analysis, and potential chimeric reads were discarded by using the UCHIME chimera slayer (Edgar et al., 2011) in RDPipeline. To compare the microbial diversity among the soil samples, the bacterial and archaeal read numbers of each sample were normalized to those of the sample with the smallest number of reads by random removal of sequencing reads using the sub.sample command of the Mothur program (Schloss et al., 2009). Operational taxonomic units (OTUs), Shannon–Weaver (Shannon and Weaver, 1963), and Chao1 biodiversity (Chao, 1987) indices and evenness for the normalized sequencing reads were computed with RDPipeline at a 97% identity cutoff value.

The bacterial and archaeal communities along a depth gradient in the bulk and rhizosphere soils of the rice paddy were compared using the UniFrac analysis (Lozupone and Knight, 2005). Representative sequences were selected by aligning the normalized reads and clustering them at a 97% nucleotide identity cutoff in RDPipeline, and singletons were removed as described by Zhou et al. (2011). The representative sequences without singletons were aligned in RDPipeline and neighbor-joining (NJ) trees were constructed based on the Kimura two-parameter model using the PHYLIP software (ver. 3.695) (Felsenstein, 2002). The NEXUS files of the NJ trees were used as input files for weighted hierarchical clustering and principal coordinate analysis (PCoA).

Taxonomic classification was performed for all high-quality sequences of *Bacteria* and *Archaea* at the phylum, class, and genus levels based on the SILVA database (v.102), using the nearest-neighbor method within the mothur program (Pruesse et al., 2007). The absolute abundances of methanotrophic and methanogenic groups along a depth gradient in the bulk and rhizosphere soils of the rice paddy were estimated by multiplying the relative abundances of methanotrophic and methanogenic groups that were classified at the genus level and the gene copy numbers of bacterial and archaeal 16S rRNA genes that were obtained by the qPCR analysis.

### Nucleotide sequence accession number

The pyrosequencing data of the bacterial and archaeal 16S rRNA genes are publicly available in the NCBI Short Read Archive (SRA) under accession no. SRP052852 (NCBI BioProject PRJNA273696).

## Results

### Vertical profiles of oxygen, methane, and TOC concentrations

**Figure 1:**
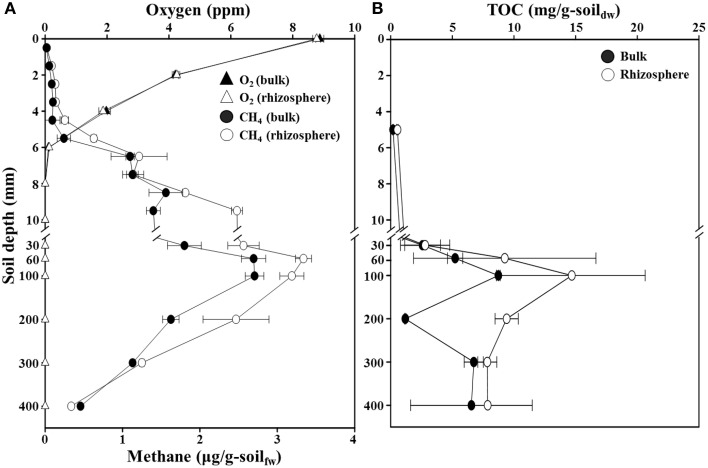
**Profiles of oxygen, methane (A), and total organic carbon (TOC) (B) concentrations along a depth gradient in the bulk and rhizosphere soil of the flooded rice paddy**. The measurements were performed in triplicate. Errors bars indicate standard deviations (*n* = 3). g-soil_fw_, gram-soil fresh weight; g-soil_dw_, gram-soil dry weight.

The concentration profiles of oxygen, methane, and TOC along a depth gradient in the flooded rice paddy are shown in Figure 1. The oxygen concentration in the surface floodwater of the paddy soil was approximately 8.8 ppm, but the concentration decreased very sharply to below the detection limit at 8-mm (Figure 1A). This result suggested that the oxic-anoxic interface representing the penetration limit of oxygen from the soil surface was located at the depth range of approximately 6–10-mm, which was slightly different from previously reported results in which the oxic-anoxic interfaces were located at approximately 2-mm depth (Revsbech et al., 1999; Reim et al., 2012). This difference might be explained by the high oxygen concentration in the floodwater in this study. No clear difference was observed in the vertical oxygen profiles of the bulk and rhizosphere soils. The methane concentrations were extremely low in the aerobic surface soil (<0.1 μg/g-soil) (Figure 1A). Simultaneous depletion of oxygen and methane in the 4–10 mm oxic-anoxic interface zone is characteristic of aerobic methanotrophy. Below this interface zone, where aerobic methanotrophy certainly occurred *in situ*, methane concentration increased steadily with depth to maximum levels of 2.7 and 3.3 μg/g-soil at 60–100-mm in the bulk and rhizosphere soils, respectively; methane then decreased to approximately 0.4 μg/g-soil at the 400-mm depth in both soils (Figure 1A). Interestingly, the methane concentration at a depth of 9 mm in the rhizosphere soil was nearly double that at the same depth in the bulk soil. The TOC concentrations increased to maximum levels of 8.7 and 14.7 mg/g-soil at approximately 100-mm depth in the bulk and rhizosphere soils, respectively (Figure 1B). The vertical profiles of TOC corresponded well with those of methane concentrations. As expected, both the methane and TOC concentrations were generally higher in the rhizosphere than in the bulk soil of the rice paddy.

### Abundances of *Bacteria* and *Archaea* along a depth gradient

**Figure 2:**
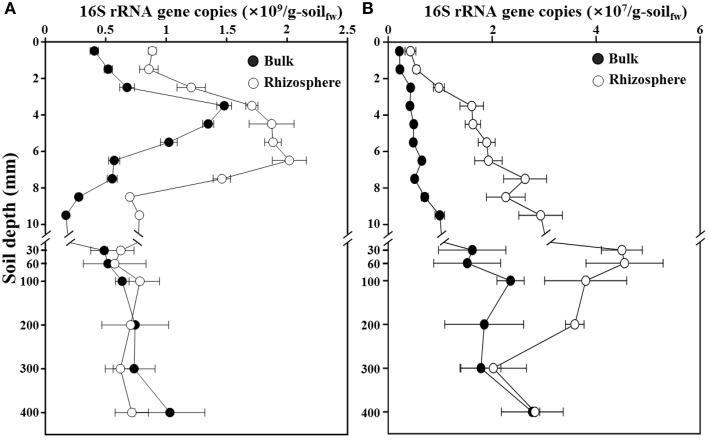
**Profiles of the 16S rRNA gene copies for total *Bacteria* (A) and *Archaea* (B) along a depth gradient in the bulk and rhizosphere soil of the flooded rice paddy**. The measurements were performed in triplicate. Errors bars indicate standard deviations (*n* = 3). g-soil_fw_, gram-soil fresh weight.

To estimate the bacterial and archaeal abundances along a depth gradient in the bulk and rhizosphere soils of the rice paddy, a qPCR approach targeting the 16S rRNA genes was used (Figure 2). The total numbers of 16S rRNA gene copies of *Bacteria* were approximately 4.1 × 10^8^ and 8.9 × 10^8^ copies/g-soil at the surface of the bulk and rhizosphere soils, respectively. The copy numbers increased sharply to a maximum of 1.5 × 10^9^ copies/g-soil at 3–4-mm depth and 2.0 × 10^9^ copies/g-soil at 7–8-mm depth in the bulk and rhizosphere soils, respectively, and then rapidly decreased to numbers similar to those at the surface, at 10-mm depth (Figure 2A). These results are consistent with the hypothesis that organic carbon, including methane, may be supporting the *in situ* growth (hence high populations) of *Bacteria* at the oxic-anoxic interface of the rice paddy. Below 10-mm depth, which corresponds to anaerobic conditions, the bacterial 16S rRNA gene copy numbers remained relatively constant at approximately 7.0 × 10^8^ copies/g-soil in both the bulk and rhizosphere soils. The 16S rRNA gene copy numbers of *Archaea* were approximately 2.2 × 10^6^ and 4.3 × 10^6^ copies/g-soil at the surface of the bulk and rhizosphere soils, respectively (Figure 2B). The copy numbers increased to a maximum of 2.4 × 10^7^ and 4.5 × 10^7^ copies/g-soil at 60–100-mm depth in the bulk and rhizosphere soils, respectively, and then gradually decreased below 100-mm depth with increasing depth in the rice paddy. The 16S rRNA gene copy numbers of *Bacteria* were higher in the rhizosphere than in the bulk soil at 0–30-mm depth, while those of *Archaea* were higher in the rhizosphere than in the bulk soil at all sampled depths.

### Bacterial and archaeal community composition along a depth gradient

**Figure 3:**
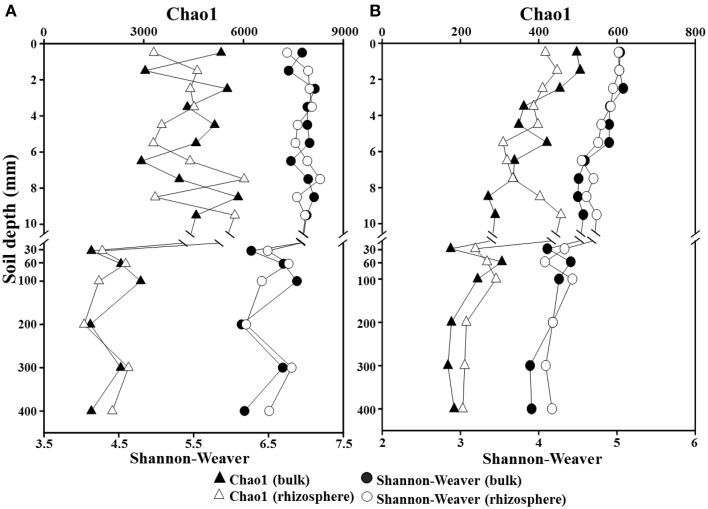
**Profiles of Chao1 and Shannon–Weaver indices for total *Bacteria* (A) and *Archaea* (B) along a depth gradient in the bulk and rhizosphere soils of the flooded rice paddy**. Both indices were computed using the normalized pyrosequencing read data at a 97% identity cutoff value.

To investigate the phylogenetic structure of the bacterial and archaeal communities along a depth gradient in the bulk and rhizosphere soils of the rice paddy, a parallel pyrosequencing approach was applied. After the removal of low-quality and putative chimeric reads, and trimming of the barcoded PCR primers, a total of 163,731 and 116,773 high-quality reads with corresponding average read lengths of 474 and 495 bases were obtained for the bacterial and archaeal communities, respectively (Supplementary Table S2). Because the read numbers influence the statistical diversity indices, especially the Chao1 and Shannon–Weaver indices, the numbers of the bacterial and archaeal reads in each sample were normalized to 3031 and 2122 reads, respectively, and the statistical diversity indices were calculated based on the normalized samples (Table S2). The Chao1 and Shannon–Weaver indices indicated that the bacterial diversities were relatively constant over the first 10-mm in both the bulk and rhizosphere soils, but they decreased below the 30-mm depth (Figure 3A), suggesting that the oxygen concentration is an important factor for bacterial diversity in the rice paddy. The archaeal diversities decreased steadily in both soils between the surface and 400-mm depth with increasing depth (Figure 3B). However, no obvious differences in the bacterial and archaeal diversities along a depth gradient were observed between the bulk and rhizosphere soils of the rice paddy.

**Figure 4:**
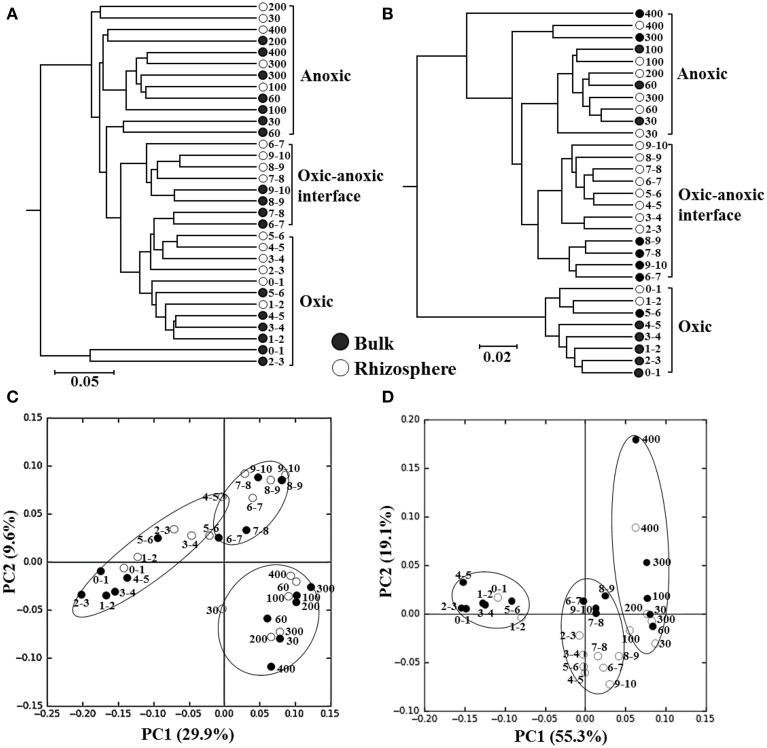
**The weighted UniFrac clustering (A,B) and PCoA (C,D) showing the relationships of bacterial (A,C) and archaeal (B,D) communities along a depth gradient in the bulk and rhizosphere soils of the flooded rice paddy**. Numbers beside the symbols represent the depth (mm) from the surface. The scale bars in the trees represent the weighted UniFrac distances.

The composition of the bacterial and archaeal communities along a depth gradient in the bulk and rhizosphere soils of the rice paddy was statistically compared using weighted UniFrac clustering and PCoA. Because Zhou et al. (2011) demonstrated that singletons can cause data distortion, these were removed from the normalized data before clustering and PCoA (Figure 4). The sequences of *Bacteria* were clustered three groups according to depths of 0–6, 6–10, and 30–400 mm (Figure 4A), corresponding with the oxic zone, the oxic-anoxic interface, and the anoxic zone, respectively, based on the vertical oxygen profiles as shown in Figure 1A. The sequences of *Archaea* were clustered into three groups according to depth; however, the clusters were less tightly related to the vertical oxygen profiles than that of the bacterial community data, especially in the oxic-anoxic interface of the rhizosphere soil (Figure 4B). The PCoA results confirmed that the bacterial and archaeal communities could be divided into three groups corresponding with the vertical oxygen profiles (Figures 4C,D). However, the bacterial and archaeal communities were not clearly differentiated between the bulk and rhizosphere soil of the rice paddy. Taken together, these results showed that the composition of the bacterial and archaeal communities of the rice paddy differed along a depth gradient and suggested that the oxygen concentration might be a determining factor in this.

**Figure 5:**
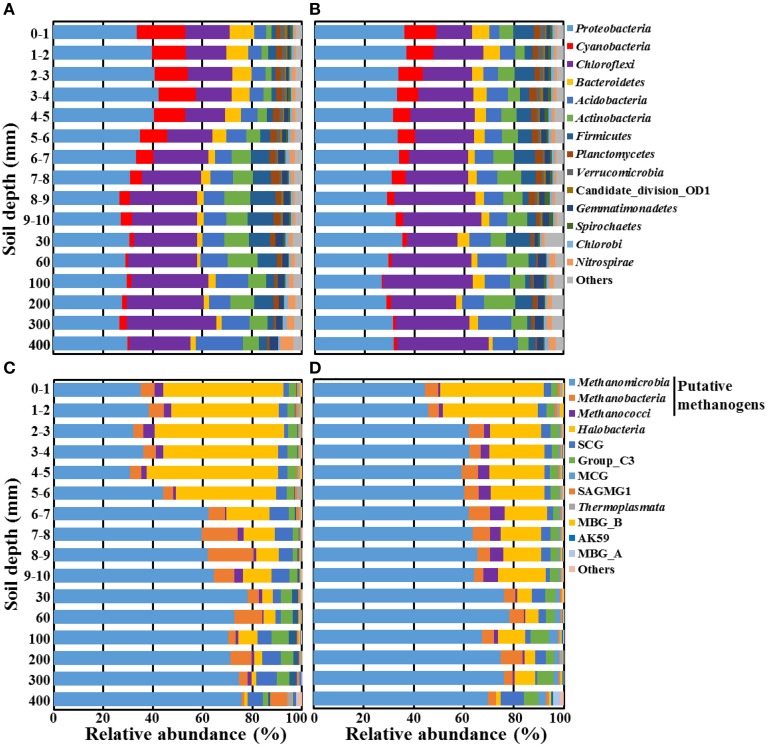
**Taxonomic compositions of bacterial (A,B) and archaeal (C,D) communities along a depth gradient in the bulk (A,C) and rhizosphere (B,D) soils of the rice paddy**. The bacterial and archaeal 16S rRNA gene sequences were classified at the phylum and class levels, respectively, by using the mothur program based on the SILVA database. “Others” represents taxa that comprised <1 and 0.1% of the total reads in all of the bacterial and archaeal samples, respectively. SCG, Soil_Crenarchaeotic_Group; MCG, Miscellaneous_Crenarchaeotic_Group; SAGMG1, South_Africa_Gold_Mine_Goup_1; MBG, Marine_Benthic_Group.

Next, the high-quality bacterial and archaeal sequences were taxonomically classified at the phylum and class levels, respectively, using the SILVA reference database (Figure 5). The classification showed that the bacterial and archaeal communities were not distinctly differentiated between the bulk and rhizosphere soils of the rice paddy. Predominant *Bacteria* belonged to the phyla, *Proteobacteria*, *Cyanobacteria*, *Chloroflexi*, *Bacteroidetes*, *Acidobacteria*, *Actinobacteria*, and *Firmicutes* at all depths in both the bulk and rhizosphere soils (Figures 5A,B), although their relative abundances were slightly different along a depth gradient. The relative abundances of *Cyanobacteria* and *Bacteroidetes* decreased, while those of *Chloroflexi*, *Acidobacteria*, and *Actinobacteria* increased with depth in both soils. The taxonomic classification of the archaeal sequences showed that methanogens, including *Methanomicrobia*, *Methanobacteria*, and *Methanococci*, which belong to the phylum *Euryarchaeota* were predominant at all depths in both the bulk and rhizosphere soils (Figures 5C,D). The relative abundance of the methanogens, especially *Methanomicrobia*, increased with depth, while non-methanogenic *Halobacteria*, which were predominant at the surface layer, sharply decreased with depth and formed a minority at the deep soil layer. These results suggested that *Halobacteria* in the rice paddy are aerobic, which was consistent with previously reported results that archaeal isolates belonging to *Halobacteria* are aerobic (Wainø et al., 2000; Shimane et al., 2011). In addition, Soil_Crenarchaeotic_Group (SCG) and Group_C3 belonging to the phylum *Crenarchaeota* were detected at high relative abundance and their relative abundances gradually increased with depth in both the bulk and rhizosphere soils (Figures 5C,D). The changes in the bacterial and archaeal communities were more pronounced at the surface layer (~10 mm) associated with a sharp oxygen gradient, suggesting that oxygen might be a determining factor for the taxonomic composition of the microbial communities.

**Figure 6:**
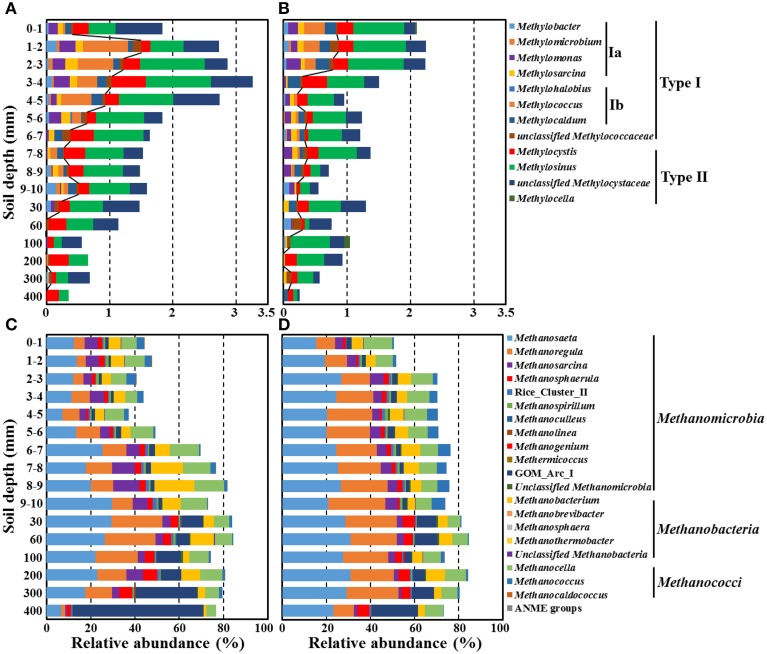
**Relative abundances of putative methanotrophs (A,B) and methanogens (C,D) classified at the genus level along a depth gradient in the bulk (A,C) and rhizosphere (B,D) soils of the rice paddy**. The lines in **(A,B)** indicate the boundaries between type I (left) and type II (right) methanotrophs. GOM_Arc_I; Gulf of Mexico_Archaea_I.

**Figure 7:**
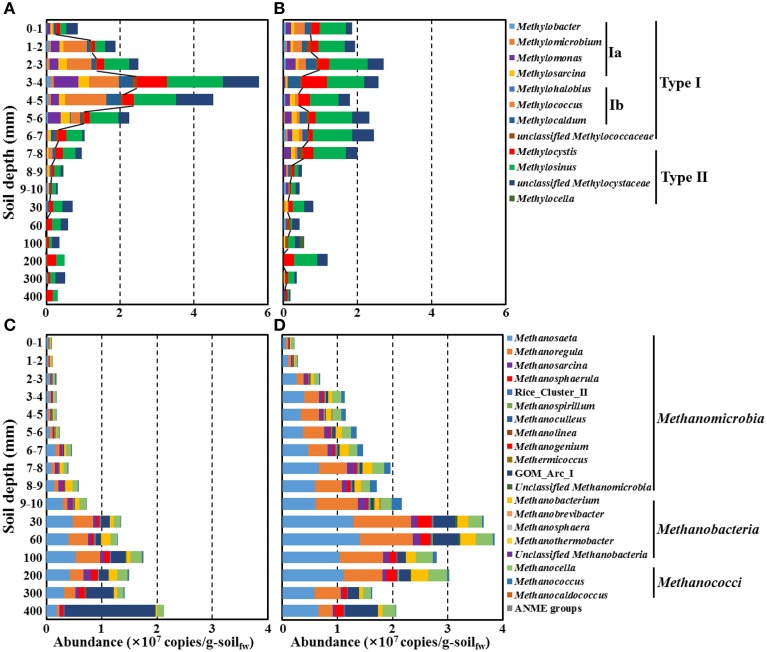
**Estimated absolute abundances of putative methanotrophs (A,B) and methanogens (C,D) along a depth gradient in the bulk (A,C) and rhizosphere (B,D) soils of the rice paddy**. The absolute abundances (16S rRNA gene copy numbers) of methanotrophs and methanogens were estimated by multiplication of the relative abundances in Figure 6 and the corresponding 16S rRNA gene copy numbers of *Bacteria* and *Archaea* in Figure 2. The lines in **(A,B)** indicate the boundaries between type I (inside) and type II (outside) methanotrophs. g-soil_fw_, gram-soil fresh weight.

We sought deeper insights into the paddy soil microbial communities by moving from phylum- and class-level analyses (Figure 5) to the more rarified genus level. Here we focused on bacterial and archaeal sequences specifically involved in methane metabolic processes—methanogens and aerobic methanotrophs along the depth gradient in the rice paddy soil (Figures 6, 7). Only 0.25–3.27% of the bacterial sequences were assigned as methanotrophs, while 37.3–88.1% of the archaeal sequences were recognized as methanogens (Figures 6A–D, respectively); these proportions of methanotrophs and methanogens were consistent with previously reported results (Lee et al., 2014). The relative abundances of the methanotrophs in the bulk soil increased in the surface layer and their maximum abundance was observed at 3–4-mm depth, but they gradually decreased with depth (Figure 6A). Whereas, the relative abundances of the methanotrophs in the rhizosphere soil showed a maximum level at the surface layer (1–3 mm) and then gradually decreased with depth (Figure 6B). Members of *Methylosinus*, *Methylocystis*, *Methylococcus*, *Methylomonas*, and *Methylosarcina* were detected as dominant methanotrophs residing in both the bulk and rhizosphere soils, and their relative abundances differed along a depth gradient. The relative abundance of type II methanotrophs was nearly constant or decreased slowly with depth in both the bulk and rhizosphere soils, while that of type I methanotrophs, in particular the type Ib *Methylococcus*, decreased more sharply with depth. In contrast to the methanotrophs, the relative abundances of the methanogens increased with depth, and a pronounced increase was observed around the oxic-anoxic interface (Figures 6C,D). *Methanosaeta*, *Methanoregula*, *Methanocella*, *Methanosarcina*, *Methanobacterium*, *Methanosphaerula*, and GOM_Arc_I (Gulf of Mexico_*Archaea*_I) were the dominant methanogens in both soils, and their relative abundances differed along a depth gradient. The relative abundances of the genera *Methanosaeta*, *Methanoregula*, *Methanosphaerula*, and GOM_Arc_I, which belong to the class *Methanomicrobia*, increased with depth. In particular, GOM_Arc_I was one of the predominant members at the deep soil layer (400 mm). Species of the ANME group were hardly detected at all depths of the rice paddy, which was consistent with previously reported results (Lee et al., 2014).

Trends in the rice paddy methane profile in Figure 1A (drastic methane depletion at the 4–10 mm depths in both bulk and rhizosphere soils and a methane concentration at 9 mm in the rhizosphere soil nearly double that that of the bulk soil) were not readily interpretable based on the relative abundances of methanotrophs and methanogens shown in Figure 6. With the goal of identifying populations potentially involved in *in situ* methane production and consumption, we next estimated the absolute abundances (16S rRNA gene copies) of methanotrophic and methanogenic populations. To accomplish this, we multiplied the relative abundances of each group of populations with their 16S rRNA gene copy numbers (Figure 7). Absolute abundances of total methanotrophs and methanogens were ~10^7^ 16S rRNA gene copies/g-soil in both the bulk and rhizosphere soils. This result was in agreement with previously reported results, in which the abundances of methanotrophs and methanogens were analyzed by using qPCR of the methyl-coenzyme M reductase (*mcrA*) and particulate methane monooxygenase (*pmoA*) genes, which encode key enzymes of methanogens and methanotrophs, respectively (Yuan et al., 2009; Shrestha et al., 2010; Lee et al., 2014).

It is important to acknowledge that methane is a metabolic end product produced in deep soil horizons that diffuses upward toward the narrow oxic surface zone where aerobic methanotrophs flourish. Focusing on the deep soil horizons that are the source of methane (Figures 7C,D), the absolute abundances of methanogens were clearly higher in the rhizosphere than in the bulk soil above 200 mm depth; this is consistent with higher plant-derived TOC in the rhizosphere (Figure 1B) fueling fermentative food chains whose final step is mediated by methanogenic populations. Methanogenic populations clearly enriched in the rhizosphere soil at the depths of 200, 100, 60, and 30 mm depths (relative to the bulk soil) included *Methanosaeta*, *Methanoregula*, *Methanocella*, *Methanobacterium*, *Methanosphaerula*, and GOM_Arc_I; these genera are candidates for *in situ* methanogenesis that may have led to the high concentration of rhizosphere methane at the 9 mm depth (Figure 1A). The most obvious trend in the absolute abundances of the methanotrophic populations is the fact that total numbers were enriched at deeper depths (especially 6–7 and 7–8 mm) in the rhizosphere soil (Figures 7A,B). This enrichment is consistent with the notion that higher methanogenic populations in the deep rhizosphere (relative to bulk) soils lead to higher concentrations and fluxes of methane; thus, methanotropic populations likely were responding to that flux at deeper depths. Particular populations enhanced at the 6–8 mm depth (relative to the same depths in the bulk soil profile) included: *Methylosarcina, Methylococcus, Methylosinus*, and unclassified *Methylocystaceae*; these genera are candidates for *in situ* methanotrophy. It must be acknowledged that another key factor likely influencing the abundances of methanotrophs in the rhizosphere vs. soils shown in Figures 7A,B may be channels formed in the soil by rice roots and their secondary impacts on oxygen and methane distribution (Kumaraswamy et al., 1997; Eller et al., 2005; Kerdchoechuen, 2005; Gutierrez et al., 2014).

## Discussion

Methane production and oxidation are expected to vary with soil depth and methane is expected to be transported in flooded rice paddies because the soil-surface region alone is aerobic in the flooded rice paddies. However, to date, only few studies on the microbial communities along a depth gradient in rice paddies have been performed, and these did not investigate methanotrophs (Lüdemann et al., 2000; Noll et al., 2005). Recently, the bacterial methanotroph communities at the soil surface and the archaeal methanogen communities in anaerobic regions have been investigated by using T-RFLP and denaturing gradient gel electrophoresis, respectively (Watanabe et al., 2010; Reim et al., 2012). In these studies, no analysis of the concomitant methane concentrations along a depth gradient in the rice paddies was performed, which made it impossible to accurately trace the methane metabolic processes in the rice paddies. Therefore, in this study, we investigated the communities of methanotrophs as well as methanogens using parallel 454-pyroseqeuncing along a depth gradient comprising the surface and anaerobic regions, along with the analysis of oxygen, methane, and TOC concentrations and methanogenic and methanotrophic abundances, in the bulk and rhizosphere soils of the flooded rice paddy.

The methane and TOC concentrations and the methanogen abundances were clearly higher in the rhizosphere soil than in the bulk soil (Figures 1, 7), suggesting that the organic carbon for methane production are derived mainly from the root exudates of the rice plants, as reported previously (Lu et al., 2000; Kimura et al., 2004; Lu and Conrad, 2005; Yuan et al., 2012; Pump et al., 2015). Bolstering the notion that *in situ* methane production and flux was high in the rhizosphere was the observation (Figure 1A) that at 9 mm, the methane concentration in the rhizosphere soil was nearly double that at the same depth in the bulk soil. The vertical methane concentration and methanogen abundance profiles indicated that methane is maximally produced at 30–200-mm depth in the rhizosphere soil (Figures 1, 7), while the vertical oxygen concentration and methanotroph abundance profiles suggested that methane is metabolized primarily aerobically at the oxic-anoxic interface in the rice paddy, where both methane and oxygen are supplied (Figures 1, 7); ANME sequences were scarcely detected in the rice paddy soil. These results suggested that methane is produced mainly from organic carbon derived from the rice roots at 30–200-mm depth in the rhizosphere soil and diffuses into the bulk and surface soil layers, and is oxidized mainly at the oxic-anoxic interface. It has been reported that oxygen is released through the aerenchyma of plant roots and that a slightly aerobic condition can occur at the rhizosphere of rice plants (Lu et al., 2000; Shrestha et al., 2008; Blossfeld et al., 2011; Ma et al., 2013). However, in this study, no clear differences in the oxygen concentrations in the bulk and rhizosphere soils were observed (Figure 1A). The total bacterial abundances were distinctly higher in the rhizosphere soil than in the bulk soil (Figure 2A). Moreover, the relative abundance of methanotrophs in the rhizosphere soil was clearly lower than those in the bulk soil (Figures 6A,B), indicating that bacteria other than methanotrophs were enriched in the rhizosphere. These results might be explained by the exudation of organic matter from rice plant roots that increased the growth of heterotrophic bacteria, causing rapid consumption of oxygen near the rice roots.

It has been reported that methanotrophs from distinct taxonomic groups are influenced differently by oxygen and methane concentrations (Henckel et al., 2000; Lüke and Frenzel, 2011; Reim et al., 2012; Lüke et al., 2014), indicating that the methanotroph abundance and composition can differ along a depth gradient in rice paddies. The abundances of the methanotrophs were observed to peak around the oxic-anoxic interface where both oxygen and methane are present, which was consistent with the results of a previous study on methanotrophic activities based on *pmoA* transcript levels and oxygen respiration at the oxic-anoxic interface (Reim et al., 2012). The study by Reim et al. (2012) showed that an alternating pattern of predominance of type Ia and type II methanotrophs occurred at the surface and that type Ia methanotrophs were predominant around the oxic-anoxic interface. However, in the current study, no alternating pattern of type Ia and type II methanotrophs was observed around the oxic-anoxic interface, which might be explained by the less distinct stratification in oxygen and methane gradients in the planted rice paddy or by differences in environmental features. Instead, type Ib methanotrophs dominated the type Ia methanotrophs at the oxic-anoxic interface. The abundances of type I methanotrophs such as *Methylococcus*, *Methylomonas*, and *Methylocaldum* decreased more obviously compared to type II methanotrophs with increasing depth of the rice paddy, while the abundances of type II methanotrophs such as *Methylocystis* and *Methylosinus* were relatively constant along a depth gradient (Figures 6A,B), which suggests that type I methanotrophs require high oxygen availability to oxidize methane, while type II methanotrophs can oxidize methane more efficiently under limited oxygen availability. These results were consistent with those of previous studies, which showed that type I methanotrophs generally display high methane-oxidizing activity under high oxygen and limited methane availability compared to type II methanotrophs (Henckel et al., 2000; MaCalady et al., 2002; Knief and Dunfield, 2005; Shrestha et al., 2008; Wu et al., 2009).

The fact that methanogens are obligate anaerobes and that the organic carbon for methane production is derived mainly from root exudates in rice paddies may explain why the methanogen abundances gradually increased with depth and peaked in the rhizosphere soil at 30–200 mm depth (Figures 6, 7). *Methanosaeta* and *Methanoregula*, known as acetoclastic and hydrogenotrophic methanogens, respectively, were predominant in the rice paddy, which suggests that hydrogen as well as acetate are important substrates for methanogenesis in rice paddies. In particular, the abundances of *Methanoregula* increased rapidly along a depth gradient, suggesting that the hydrogen concentration increases with depth (Bräuer et al., 2006). Although the methane concentrations were very low below 300-mm depth (Figure 1A), the absolute abundances of populations classified as the methanogens were high due to the presence of GOM_Arc_I-related archaea (Figures 7C,D). Although the GOM_Arc_I sequences were classified within *Methanomicrobia* in this study, the GOM_Arc_I, which was first identified from methane hydrate-rich sediments in the Gulf of Mexico, was originally presumed as ANME-2d because their 16S rRNA gene sequences were also phylogenetically related to those of ANME-2d (Mills et al., 2003, 2005). However, to date, no study establishing physiological function of GOM_Arc_I as methanogens or ANME has been published. In the current study, the abundances of GOM_Arc_I rapidly increased with decreasing methane concentrations, which supports the notion, in rice paddy soils, the GOM_Arc_I may be heterotrophic archaea; corroborated by the high TOC concentrations below 300-mm depth (Figures 1, 7).

In this investigation, we combined the millimeter-scale sampling of oxygen, methane, TOC concentrations, together with pyrosequencing-based community characterization of communities, especially methanotrophs and methanogens, in both bulk and rhizosphere soils of a planted rice paddy. By focusing on absolute abundances of both methanogenic and methanotrophic genera (Figures 7C,D and Figures 7A,B, respectively), we found contrasts between methanogens and methanotrophs at depths sampled across rhizosphere and bulk soils that helped explain drastic methane depletion at the 4–10 mm depths in both bulk and rhizosphere soils and a methane concentration at 9 mm in the rhizosphere soil nearly double that that of the bulk soil. As a result we hypothesize that populations of methanogens (*Methanosaeta*, *Methanoregula*, *Methanocella*, *Methanobacterium*, and *Methanosphaerula*) and methanotrophs (*Methylosarcina, Methylococcus, Methylosinus*, and unclassified *Methylocystaceae*) likely were physiologically active *in situ*.

### Conflict of interest statement

The authors declare that the research was conducted in the absence of any commercial or financial relationships that could be construed as a potential conflict of interest.
