# Supplementary material for: High resolution depth distribution of Bacteria, Archaea, methanotrophs, and methanogens in the bulk and rhizosphere soils of a flooded rice paddy
Source: Front Microbiol. 2015 Jun 25;6:639. doi: 10.3389/fmicb.2015.00639 (PMC4479796; doi:10.3389/fmicb.2015.00639)
Supplement: Supplementary file 1 [file Presentation1.PDF]

SUPPLEMENTARY INFORMATION

High resolution depth distribution of *Bacteria*, *Archaea*, methanotrophs, and methanogens in the bulk and rhizosphere soils of a flooded rice paddy

Hyo Jung Lee[1], Sang Eun Jeong[1], Pil Joo Kim[2], Eugene L. Madsen[3] and Che Ok Jeon[1]

[1]*Department of Life Science, Chung-Ang University, Seoul, Republic of Korea*

[2]*Division of Applied Life Science, Gyeongsang National University, Jinju, Republic of Korea*

[3]*Department of Microbiology, Cornell University, Ithaca, NY, USA*

Supplementary Figure S1. A schematic diagram showing sampling points of bulk and rhizosphere soils in the flooded rice paddy.

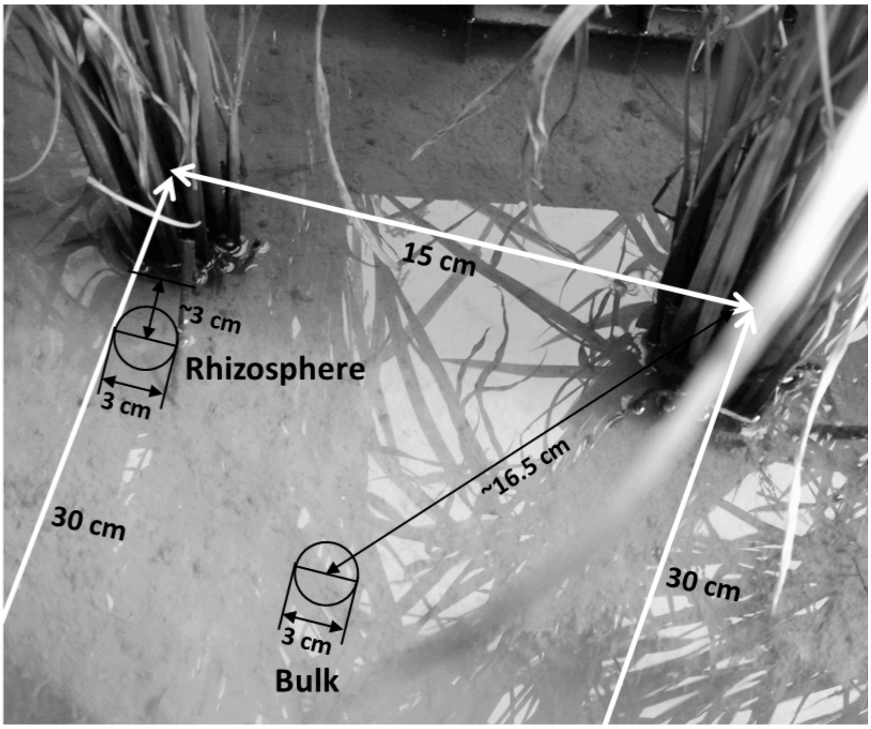

Supplementary Table S1. Sequence information of the barcodes and adapters used in this study

Name	Sequence (5´-3´)	
**Adapter sequences**		
A adapter	CCATCTCATCCCTGCGTGTCTCCGACTCAG	
B adapter	CCTATCCCCTGTGTGCCTTGGCAGTCTCAG	
**Sample***	***Bacteria***	***Archaea***
B0-1	TATGCAC	CATGCTC
B1-2	TCTGCAG	TATGCAC
B2-3	AGATCGCT	TATGCAC
B3-4	CTACACAG	AGATCGCT
B4-5	ACTACACGC	TCGCTATC
B5-6	TCGAGTAG	AGTCACTAG
B6-7	CTGTCTACG	TGACGACGT
B7-8	CAGTCTCGA	CAGTCTCGA
B8-9	TCTGTCTCGC	TCTGTCTCGC
B9-10	TACGCTCTCGT	TCATATACGCG
B30	TCGTCAT	TGTCAGC
B60	CGATGAG	AGAGCTG
B100	ATGCAGAC	TCTCGACT
B200	CATGTAGC	CACACTGA
B300	CAGTCTCGA	TGACGACGT
B400	CACTATGTG	AGTCACTAG
R0-1	AGCGATG	AGAGCTG
R1-2	TCAGATG	TCAGATG
R2-3	ATGCTGAG	CGCATATC
R3-4	ATGCAGAC	TCTCGACT
R4-5	ACACTGTG	ATGCAGAC
R5-6	CAGACAGAT	CTGTCTACG
R6-7	CAGACAGAT	ACTACACGC
R7-8	TGACTCGAC	TGACTCGAC
R8-9	TGAGTGACGC	ATCGTCTGTG
R9-10	TACGCTCTCGT	TCATATACGCG
R30	ATGTACGATG	TGAGTGACGC
R60	TCGCAGACAC	TCGCAGACAC
R100	TAGATAGTGCG	TAGATAGTGCG
R200	CTGTCTACG	ACGTCTCTACG
R300	TCGCTAGTGTA	CTAGAGACACT
R400	TACGCTCTCGT	ACACACGCATC

*B and R indicate the bulk and rhizosphere soils of the rice paddy, respectively and the numbers represent the depths (mm) from the surface.

Supplementary Table S2. Statistical analysis of the normalized 16S rRNA gene sequencing data showing the bacterial and archaeal diversities along a depth gradient in the rice paddy*

Sample†	*Bacteria**					*Archaea**				
	HQS‡	OTU†	Shannon	Chao1	Evenness	HQS‡	OTU†	Shannon	Chao1	Evenness
**Bulk**										
B0-1	5677	1886	7.17	5987	0.95	3079	313	5.04	497	0.88
B1-2	4323	1564	6.95	3905	0.95	3526	318	5.03	506	0.87
B2-3	3031	1962	7.29	6365	0.96	2498	328	5.08	454	0.88
B3-4	3489	1822	7.19	5289	0.96	2742	281	4.91	362	0.87
B4-5	4192	1821	7.18	5346	0.96	5154	270	4.90	349	0.88
B5-6	4203	1866	7.23	5543	0.96	5578	274	4.90	421	0.87
B6-7	3181	1560	6.98	3389	0.95	2621	242	4.59	338	0.84
B7-8	4574	1819	7.21	4493	0.96	3336	216	4.51	335	0.83

B8-9	5939	2030	7.33	7866	0.96	3547	213	4.50	271	0.84
B9-10	6356	1871	7.22	5262	0.96	3050	220	4.57	289	0.85
B30	4015	1169	6.65	2028	0.94	5893	175	4.11	175	0.80
B60	6072	1402	6.82	2920	0.94	3633	208	4.41	306	0.83
B100	5755	1595	7.07	3342	0.96	2956	178	4.26	244	0.82
B200	3922	1060	6.49	1835	0.93	3342	157	4.18	177	0.83
B300	6414	1401	6.85	2679	0.95	2909	145	3.89	168	0.78
B400	3330	932	6.29	1536	0.92	2720	139	3.91	184	0.79
**Rhizosphere**										
R0-1	3997	1543	6.91	3415	0.94	2122	293	5.02	417	0.88
R1-2	5070	1855	7.21	5225	0.96	2326	313	5.03	447	0.88
R2-3	6352	1887	7.25	5809	0.96	3840	301	4.95	410	0.87
R3-4	3061	1931	7.30	5598	0.96	2845	294	4.92	387	0.87
R4-5	4173	1682	7.09	4132	0.95	3904	269	4.80	398	0.86
R5-6	5189	1626	7.06	3636	0.96	2759	238	4.76	309	0.87
R6-7	5865	1836	7.21	5184	0.96	3096	230	4.55	319	0.84
R7-8	7445	2070	7.40	7000	0.97	5020	246	4.70	334	0.85
R8-9	8660	1630	6.96	4150	0.94	5548	244	4.61	403	0.84
R9-10	4526	1879	7.20	6214	0.96	5993	271	4.74	457	0.85
R30	4207	1140	6.64	2062	0.94	3704	189	4.33	238	0.83
R60	5127	1489	6.96	3117	0.95	3305	181	4.08	268	0.78
R100	3509	1071	6.52	2093	0.93	2942	212	4.43	291	0.83
R200	6245	1218	6.68	2093	0.94	3200	172	4.18	215	0.81
R300	7938	1548	7.00	3190	0.95	2849	159	4.09	211	0.81
R400	7894	1272	6.72	2273	0.94	6736	170	4.17	206	0.81

*Bacterial and archaeal diversity indices were computed using 3031 and 2122 normalized reads, respectively.

†The numbers in the sample codes represent the depth (mm) from the surface.

‡ HQS, high-quality sequences.

†OTU, operational taxonomic units.
